# A Rare Compressive Spinal Thoracic Tumor in a Young Girl Who Presented With Rapid Progressive Lower Limb Weakness

**DOI:** 10.7759/cureus.86117

**Published:** 2025-06-16

**Authors:** Sara Aljar, Raafat Hamad Seroor H Jadah

**Affiliations:** 1 General Practice, Bahrain Defence Force Hospital, Riffa, BHR; 2 Pediatric Neurology, Bahrain Defence Force Hospital, Riffa, BHR

**Keywords:** lower limb, neurofibromatosis, spinal schwannoma, spinal tumor, weakness

## Abstract

Spinal schwannoma is an uncommon condition in children, particularly when located in the thoracic region. Affected patients usually exhibit gradually worsening motor weakness, pain, and bladder control issues due to the tumor's impact on spinal nerves. These symptoms depend on the size and location of the tumor, as well as the compression of the surrounding structure. Magnetic resonance imaging (MRI) of the spine is the preferred method for diagnosing spinal schwannoma. It provides detailed information about the tumor's size, exact location, and its association with the spinal cord and nerve roots. Surgery is the primary and most effective treatment for individuals with spinal schwannoma. Due to its vague neurological symptoms, diagnosing this rare compressive tumor is very challenging, which can lead to significant delays in management. The majority of patients who have this rare spinal tumor surgically removed in the early stages of symptoms tend to experience positive outcomes. Herein, we report the case of an eight-year-old girl who presented with progressive lower limb weakness over a duration of three weeks associated with severe lower back pain, with significant loss of her motor function and bladder dysfunction. Upon presentation to the hospital, a spinal MRI showed significant compressive spinal schwannoma. She underwent urgent surgical excision of her tumor with complete recovery of her motor function upon follow-up at a pediatric neurology clinic. The aim of reporting this case was to highlight the importance of early diagnosis of this rare tumor type in pediatric patients, as well as early surgical intervention to achieve a good outcome.

## Introduction

Spinal schwannoma is a highly sporadic form of benign spinal tumor in children, with an incidence of 2.5% to 4% [1]. The most common clinical presentation of spinal schwannoma in the pediatric age group includes weakness, numbness, and paresthesia. Early diagnosis of spinal schwannoma is crucial, as they may progress and cause serious damage to the spinal cord, potentially causing paralysis. Therefore, obtaining a detailed history and performing a physical examination along with labs and spinal magnetic resonance imaging (MRI) are critical steps to diagnosing this progressive disease. It is important to highlight that the gold standard for diagnosing schwannoma is through a spinal MRI [2]. Total surgical resection, which is the treatment of choice for spinal schwannoma, is essential to ensuring the best outcome for the patient, as it is characterized by an excellent prognosis [3].

## Case presentation

An eight-year-old previously healthy female presented to the pediatric neurology clinic with a three-week history of progressive lower limb pain and weakness. The weakness had increased in severity over the course of three weeks, which was followed by episodes of frequent falls and bladder dysfunction. The patient denied numbness, fever, weight loss, or night sweats. There were no reported changes in vision, taste, or speech, as noted by the patient or her parents. The patient did not experience vomiting or any change in bowel habits and had no history of recent illness, sick contact, recent travel, or trauma. The patient was not taking regular medication. During the three weeks of illness, the patient visited multiple physicians and was diagnosed with viral myositis. She was referred to a physiotherapist, but her condition did not improve.

The patient’s perinatal history was uneventful; she was vaccinated up to her age. Growth and developmental milestones were appropriate for her age. Her parents were non-consanguineous, with no similar condition observed in the family. Past medical and surgical histories were unremarkable.

Upon physical examination, the patient was conscious, alert, and oriented. She had no dysmorphic features or neurocutaneous lesions. Neurological examination revealed normal motor function in the upper limbs. In the lower limbs, muscle tone was preserved; however, motor strength was reduced to 3/5 bilaterally, with brisk deep tendon reflexes (4+) and the presence of ankle clonus. Her cranial nerves were intact, with no cerebellar signs. The sensory examination was normal. There was no tenderness of the spine, but her range of motion was limited. She had an unsteady gait with heel walking, and she was unable to bear her own weight due to severe lower limb pain. The rest of her systemic examination was unremarkable.

**Figure 1:**
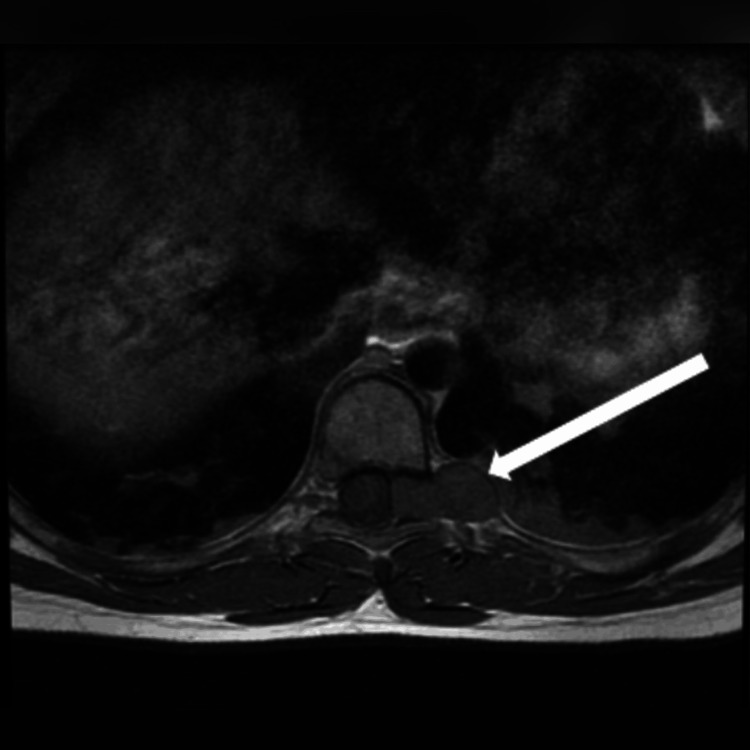
T1-weighted axial MRI showing a thoracic spinal schwannoma (arrow).

**Figure 2:**
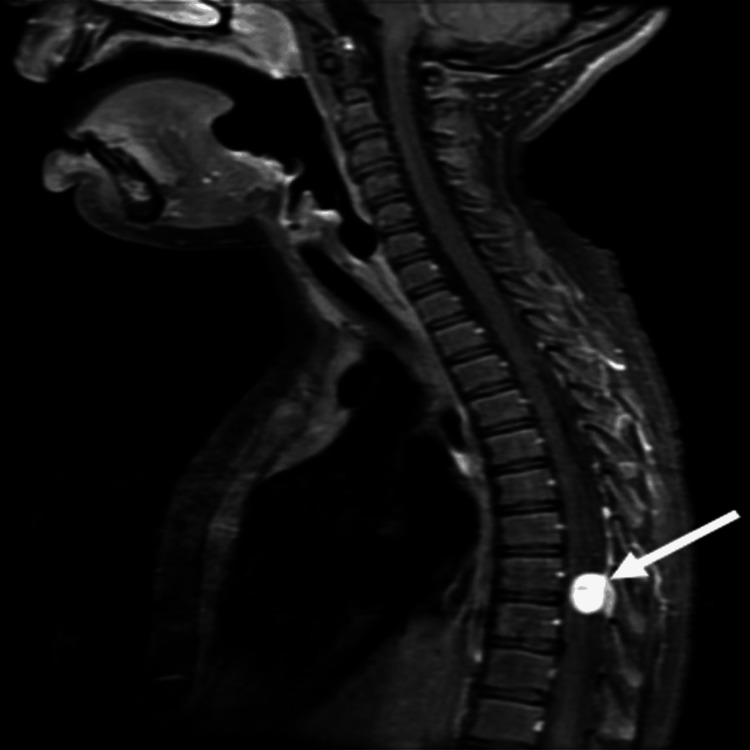
Spine MRI (T1 sagittal view) showing hypersignal intensity within the tumor site (arrow).

**Figure 3:**
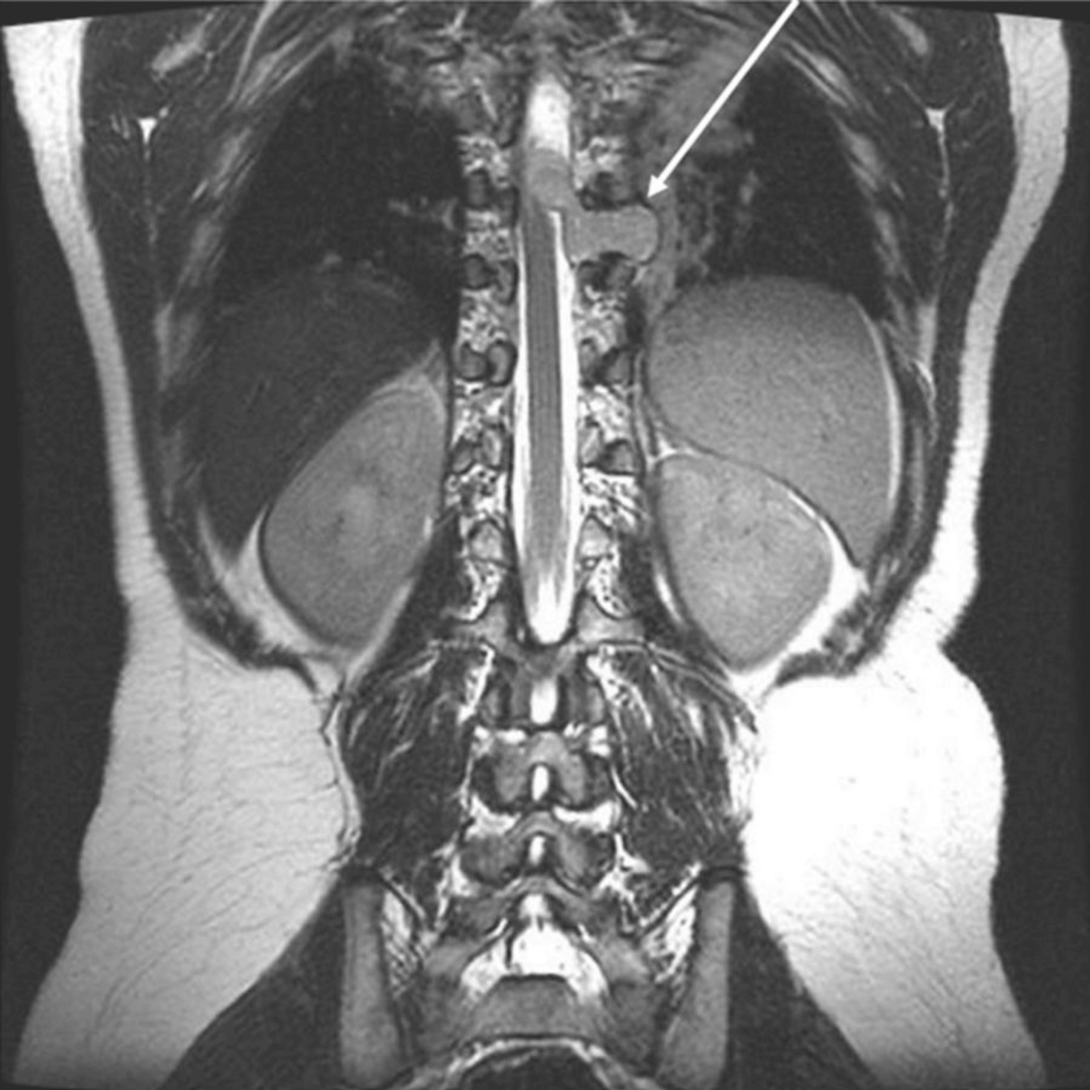
Spine MRI (T2 coronal view) showing extension of the tumor within the spinal canal with significant spinal cord compression (arrow).

Hematological and biochemical labs, including creatinine kinase, were done and were within normal ranges. Based on the patient’s clinical condition, she underwent an urgent whole-spine and brain MRI, which revealed a normal brain MRI and significant spine and nerve root compression (Figures 1-3).

**Figure 4:**
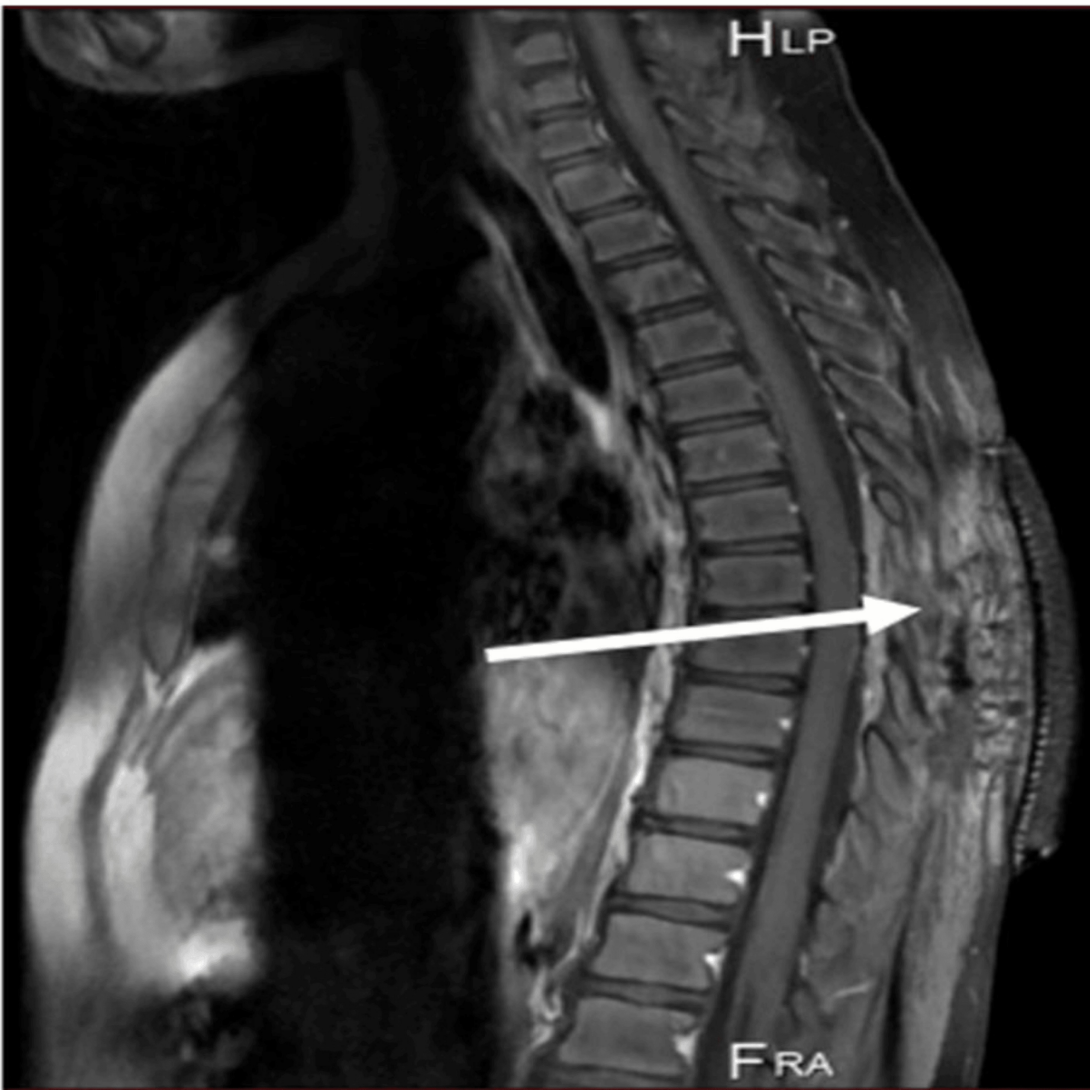
Spine MRI (T1 sagittal view) showing post-surgical excision of the tumor (arrow).

**Figure 5:**
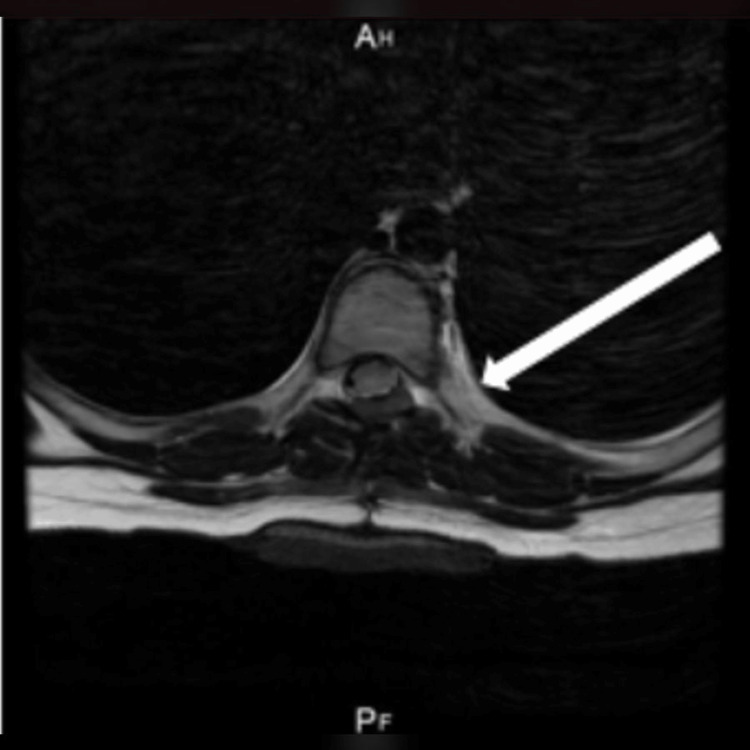
Spine MRI (T2 axial view) showing location where tumor was surgically removed (arrow).

**Figure 6:**
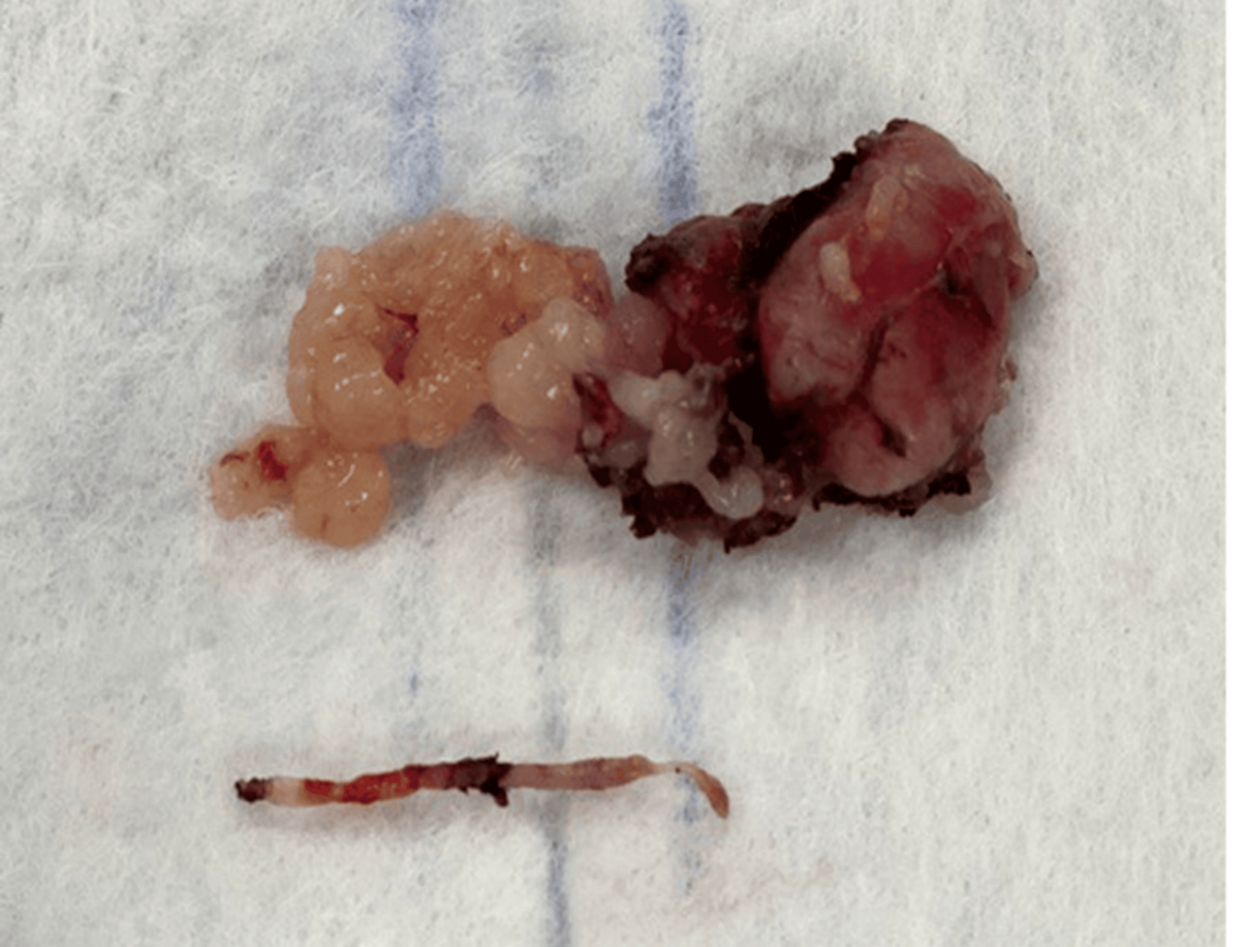
Tumor specimen removed during the surgical procedure.

The whole exome sequencing service was sent to rule out neurofibromatosis type 2 (NF2), with the report confirming a negative result. The patient underwent total surgical excision of the spine schwannoma, which led to a complete recovery (Figures 4-6). She was able to regain full power in her lower limbs and walk independently with no pain. Video 1 shows a complete surgical excision of the spinal schwannoma.

**Video 1:** Complete surgical excision of spinal schwannoma.

## Discussion

Spinal schwannoma is a slow-growing, mostly noncancerous disease that usually presents in the cervical or lumbar region, rarely in the thoracic spine [1]. Clinical symptoms can often be unclear and difficult to interpret. Many children initially exhibit non-specific signs that gradually worsen over time. Studies have shown that patients usually present with a gradual decline in motor function, reported in 86% of patients, while 67% experienced localized pain. [4]. Autonomic dysfunction, including bowel and bladder disturbances, is less common, with reported incidences ranging from 10% to 20%. [4]. To diagnose spinal schwannoma, physicians should initially take a full medical history, perform a physical examination, and conduct neuroimaging. MRI remains the gold standard for diagnosing spinal schwannomas due to its superior ability to outline tumor margins, assess spinal cord compression, and evaluate relationships with adjacent nerve roots [5]. It plays an important role in surgical planning by accurately defining the extent and anatomical features of the lesion.

In some cases, especially in pediatric patients, further genetic evaluation may be warranted to rule out syndromic associations such as neurofibromatosis type 2 (NF2), which is commonly associated with multiple nervous system tumors, including schwannomas [6]. In our case, whole-exome sequencing confirmed the absence of NF2 mutations, supporting the diagnosis of a sporadic schwannoma [6].

Surgical intervention, typically via a posterior approach with total laminectomy, is the treatment of choice for spinal schwannomas. The majority of patients who undergo surgical removal demonstrate favorable outcomes [7].

Postoperative prognosis is influenced by factors such as tumor size, anatomical location, and the patient’s neurological status before surgery. However, in general, the prognosis of spinal schwannoma is usually excellent (95% of cases) [8], and achieving full recovery is expected.

In 1995, Seppälä et al. studied the long-term outcomes of patients who underwent surgical resection of spine schwannoma; 20% of cases showed complete recovery over a follow-up period of 2.9 years. However, disease progression was noted in 21% of cases, including cystic myelopathy (2%), spinal arachnoiditis (6%), spinal deformity (6%), and complaints of pain (7%) [9].

## Conclusions

Spinal schwannoma is an extremely uncommon benign tumor type in pediatrics, especially in the thoracic region. These patients often present with clear neurological symptoms, including pain, weakness, and difficulty bearing weight. Diagnosis usually is confirmed with neuroimaging (i.e., MRI). MRI is critical for determining the exact location and extent of the tumor and serves as a valuable tool for postoperative assessment. As early surgical intervention is important to ensure full recovery, spinal schwannoma should be suspected in any patient presenting with weakness, and an MRI should be obtained. Finally, early surgical resection is crucial to prevent further neurological deterioration, halt disease progression, and improve long-term quality of life.
